# COVID-19 Vaccination Hesitancy or Acceptance and Its Associated Factors: Findings from Post-Vaccination Cross-Sectional Survey from Punjab Pakistan

**DOI:** 10.3390/ijerph19031305

**Published:** 2022-01-24

**Authors:** Rubeena Zakar, Ain ul Momina, Sara Shahzad, Mahwish Hayee, Ruhma Shahzad, Muhammad Zakria Zakar

**Affiliations:** 1 Department of Public Health, Institute of Social and Cultural Studies, University of the Punjab, Lahore 54590, Pakistan; ruhmabshahzad@gmail.com; 2 Health Service Delivery in Punjab, King Edward Medical University and Oxford Policy Management, Lahore 54000, Pakistan; ainmomina@gmail.com; 3 Department of Public Health and Primary Care, University of Cambridge, Cambridge CB2 1TN, UK; sara.cheema.37@gmail.com; 4 Oxford Policy Management, Islamabad 44000, Pakistan; Mahwish.hayee@opml.co.uk; 5 Vice Chancellor Office, University of Okara, Okara 56300, Pakistan; mzzakir@yahoo.com

**Keywords:** COVID-19, vaccination, hesitancy, acceptance, access to mass media, chronic diseases, allergies, lifestyle factors, self-reported health status

## Abstract

COVID-19 has posed massive challenges related to health, economy, and the social fabric of the entire human population. To curb the spread of the virus, the Government of Pakistan initiated a vaccination campaign against COVID-19. The objective of this research was to assess the factors associated with COVID-19 vaccine acceptance or hesitancy. The data were collected telephonically using a cross-sectional survey design through a close-ended structured questionnaire from a sample of 1325 vaccinated and non-vaccinated individuals with a response rate of 38%. SPSS v. 26 was used to analyze the data. The study revealed that 73% of the respondents were male, half in the 40–49 age group, 78% living in urban areas, and 45% had a monthly income between 20,001–50,000 Pakistani rupees. People felt reluctant to get vaccinated because of myths and misinformation related to it. The socio-demographic factors including male, age 60–69, middle or higher level of education, marital status, currently employed, from middle socio-economic status, living in urban areas, high access to mass media, history of influenza vaccination, physical activity, and perceived good health status were significantly associated with COVID-19 vaccination uptake. Concerted efforts are needed to achieve vaccine targets for the broader population through understanding and identifying barriers to vaccination.

## 1. Introduction

COVID-19 was declared a “pandemic” by the World Health Organization (WHO) in March 2020, and has posed massive challenges related to health, economy, and the social fabric of the entire human population [1]. To curb the spread of the virus, the Government of Pakistan, similar to other countries, issued several public health guidelines, preventative measures, and made widely available a solution in the form of vaccination. In Pakistan, with more than 1.15 million confirmed cases of COVID-19, around twenty-five thousand deaths as of 30 August 2021 [2], and the presence of the concern of variants in the ongoing wave of COVID-19, it is necessary to enhance vaccine coverage in the country in order to acquire herd immunity.

Following the initiation of the push for vaccination campaign in most developed and developing nations, Pakistan launched its vaccination campaign for frontline workers in February 2021 with the Chinese vaccine, Sinopharm. Currently, there are seven different vaccines including Sinopharm, Sinovac, AstraZeneca, Cansino, Sputnik, Pfizer-BioNTech and Moderna, which are registered for use in Pakistan [3]. Due to the limited production as well as limited availability of vaccine doses in the initial stages of the vaccination campaign, elderly people, healthcare workers, and other frontline individuals were prioritized for vaccination, as they were deemed most susceptible to get infected. The roll out of the vaccine then followed a decreasing age order [4] or decreasing susceptibility of catching the virus. Following this mechanism of vaccination, experts expected to vaccinate a large number of the population in a short period of time.

In Pakistan, 1% of the population was fully vaccinated and 2.6% was partially vaccinated on 3 June 2021. However, these percentages rose over a period of 3 months, and on the 25th of August, 6.3% of Pakistan’s population was fully vaccinated and 10.8% was partially vaccinated. At the time of the study, only 6.3% of 67 million of the target population of Punjab was vaccinated against COVID-19 [5]. According to epidemiologists, achieving herd immunity is impossible without 70% of the total world’s population fully immunized with the COVID-19 vaccine [6].

### 1.1. Vaccine Hesitancy and Acceptability

One of the biggest challenges in vaccine uptake is vaccine hesitancy [7]. Poliomyelitis, a vaccine-preventable disease, is eradicated globally but Pakistan is one of the only two countries in the world where it is still endemic [8]. This highlights the reluctance of people towards not only COVID-19 vaccines, but vaccines in general. A range of religious, cultural, social, and ethnic beliefs may influence the public’s decision to get vaccinated [9,10,11]. In addition to this, mistrust in government institutions [12], safety concerns and potential risks are considered an important factor in determining the vaccines’ acceptability [13]. One of the major reasons of vaccine hesitancy among people is the perceived side effects attached with receiving a dose [14]. Those who are hesitant in getting immunized believe that the rapidly developed vaccines may have alleged harms and unforeseeable adverse reactions [15], ranging from minor pain at the site of the injection to severe health conditions such as blood clotting [16,17].

### 1.2. Barriers to Vaccine Acceptance

The effectiveness of inoculation campaigns for curbing the spread of the pandemic is not only dependent on the vaccine’s safety and efficacy, but also on its availability and people’s attitude towards them [18].

According to Fisk [19], there are two types of barriers that hinder the uptake of vaccines: structural and attitudinal. Structural barriers are the systematic issues which impact an individual’s ability to access a service. They include availability- and affordability-related factors, such as cost, outlet location, or transportation. Attitudinal barriers are the perceptions or beliefs that influence the willingness of individuals that are at-risk to seek out and accept a service. They include perceptions about communicable diseases, perceptions about vaccines, and fear and trust issues with healthcare and Government agencies and satisfaction with the provided services. Of all attitudinal barriers, public trust is particularly important [19].

Currently, in Punjab, the population of age 17 and above is being registered for vaccination. A large number of vaccination centers have also been established in public schools and colleges for better outreach and access (several were operating 24 h a day and seven days a week in the initial months of the vaccination campaign). Currently, there are 677 COVID-19 Vaccination Centers (CVCs) being operated in Punjab, however, only 17.16% of the population of the country is vaccinated with at least one dose. The 24/7 availability of free vaccines and vaccination centers shows that structural barriers are not restricting Pakistan in achieving full vaccination coverage against COVID-19.

The low uptake can, thus, be linked to attitudinal barriers at the community level. In Pakistan, behavioral and communication strategies such as broadcasting vaccination messages through mobile phone ringtones, at a broader level, and through the engagement of celebrities and Government leaders who share their own vaccination experiences and general information on the vaccine [20], as well as announcements in residential areas and mosques have been used to motivate masses for immunization against COVID-19 [21].

As discussed earlier, various religious factors and misinformation circulating on social media platforms continue to influence the uptake of the COVID-19 vaccine in Punjab. Being the largest province in Pakistan, Punjab is at a high risk of COVID-19, with 388,297 confirmed cases up to 27 August 2021. Despite the Provincial Government’s campaign and efforts to promote access to the vaccine, not enough people are getting themselves vaccinated.

This paper is a part of a broader post-vaccination survey on COVID-19. The objectives of this research were to find out the attitude of people towards COVID-19 vaccination and to assess the factors associated with COVID-19 vaccine acceptance or hesitancy in the Punjab province, which houses half of Pakistan’s population. The results of this survey may be helpful in providing a data-driven solution to achieve vaccine targets for the broader population (including the most vulnerable groups), vaccine roll out, and monitoring of vaccine accessibility for all.

## 2. Materials and Methods

Due to limitations in conducting face-to-face research during the fourth wave of the COVID-19 outbreak in Pakistan from July to September 2021, telephonic interviews were conducted by enumerators using a cross-sectional survey design. Telephone was selected as the medium for data collection because it is one of the most widely used, easily accessible and low-cost platforms for communication in Pakistan [22]. The study was carried out at the provincial level and samples were collected from almost all cities of Punjab where vaccination centers were established preceding the survey. The data were collected from both vaccinated and non-vaccinated individuals above the age of 40 years.

### 2.1. Inclusion Criteria

For vaccinated group, the inclusion criteria were individuals 40 years and above who were completely vaccinated with any of the COVID-19 vaccine and/or partially vaccinated with COVID-19 vaccine AstraZeneca (the gap between the two doses of AstraZeneca is 84 days and it was first inoculated in April 2021 in Pakistan. Thus, we assumed that a majority of the recipients of this vaccine would be partially vaccinated at the time of data collection for this study).

For non-vaccinated group, the inclusion criteria were individuals aged 40 and above who either registered for the vaccine and received a vaccination date but still did not receive the dose or did not register and/or did not receive a single dose of any COVID-19 vaccine. Individuals of age 40 and above were targeted because the vaccine roll out in Punjab began with those aged 60 and above, followed by age 50 and then 40-year-olds. At the time of the survey, it was safe to assume that several candidates who had completed two doses of COVID-19 vaccination could be found in this target population. On the other hand, for non-vaccinated individuals above 40 years of age, it was assumed that they might have perceived any barrier in getting vaccination. Respondents who were approached but refused to participate, or had a serious mental or physical disease, were excluded from the survey.

### 2.2. Data Collection Tool

For data collection, a structured questionnaire was developed on the basis of extensive review of relevant literature, stakeholders’ consultations, and appraisal of misinformation about COVID-19 circulating on social media. The questionnaire was developed in English, but to minimize the communication barrier, it was translated into national language (i.e., Urdu). The questionnaire was pre-tested to identify redundant questions, determine the time required to complete the survey questionnaire and gave an idea about the response rate. The questionnaire was revised and improved in the light of pre-testing experiences as well as according to the suggestions of the experts. The final questionnaire had six sections, each with various close-ended questions (here only three sections are mentioned which are relevant to the present paper).

The first section of the questionnaire records the socio-demographic profile including age, sex, religion, highest level of education, monthly family income, type of occupation, sector of employment, occupation, region, marital status, number of children, and exposure to different forms of media i.e., television, mobile phone, newspapers and internet to seek information. Usage of one media source for seeking information was recoded as ‘low access’, two or three as ‘moderate access’, and four or more as ‘high access’.

The second section focused on co-morbidities and health status of individuals. The first question, a checklist, asked the respondent about history of various health conditions while other questions inquired about allergies, adverse events due to food, medicine, and any vaccination, including the practice of behaviors such as use of tobacco and exercise pattern [23]. The questions regarding past history of rejecting any other vaccine and if they received vaccination against influenza were also asked [24]. The next three questions in this section measured previous infection of COVID-19, testing and intensity of the illness [25]. The section concluded by assessing current health status through a rating scale ranging from ‘very poor’ to ‘very good’ [24].

The third section made use of seven questions to determine the acceptability rate of COVID-19 vaccination. The respondent was asked whether he/she is vaccinated or not and about the factors involved in the decision to get or not to get vaccinated [10,11,12,26,27]. In case the respondent is not vaccinated, he/she is inquired about willingness to get vaccinated.

### 2.3. Sample Size, Sampling Technique and Recruitment of Respondents

Sample size of the survey was calculated as 1067 on the basis of a previous study on the acceptance of COVID-19 vaccination in Southeast Asia [28]. The sample size was calculated using the formula *n* = (z^2^ × *p*q)/e^2^ with a 3% margin of error, 95% confidence interval and a 50% vaccine acceptability. Where:

- e is the desired level of precision (i.e., the margin of error),
- *p* is the (estimated) proportion of the population that has vaccination acceptability,
- q is 1 − *p*

Assuming non-response rate of 20% (based on previous research carried out by Azoulay [29], the sample size was increased to 1284 before starting the data collection. Since one of the key objectives of this study was to determine the perceived effectiveness of COVID-19 vaccination in the province, a relatively larger sample of vaccine recipients (60%) was included, as compared to vaccine non-recipients (40%).

To receive the COVID-19 vaccine in Punjab, individuals need to register themselves with the National Database and Registration Authority (NADRA). Data related to Government vaccinations is fed into this database in Punjab by the Primary and Secondary Healthcare Department (P&SHD). Hence, the list of vaccine recipients was obtained from the P&SHD.

Regarding non-vaccinated individuals, the Department maintained a limited list of those who registered for the vaccine but had not received it yet. Convenient sampling was used to generate a database of non-vaccinated individuals from all the contacts (vaccinated and non-vaccinated) provided by P&SHD. Additional contacts were collected from public sector employees including lady health workers (LHWs), lady health supervisors (LHSs) and lady health visitors (LHVs) of respective districts, individuals working under Social Security Department, workers of different factories and private organizations to generate a database of non-vaccinated individuals above 40 years of age.

From the generated databases of both vaccinated and non-vaccinated individuals, survey respondents were recruited through random sampling using computer-generated random numbers. The lists were first divided based on districts of the potential respondents to ensure that each district in the Province was represented in the sample, and then it was further divided based on the type of vaccine, so that each vaccination type was included. After this, numbers were selected using the Random Number Function (RAND) in Microsoft Excel (Microsoft Corporation, Albuquerque, NM, USA). Enumerators contacted the respondents through their given phone/mobile numbers, introduced the study to them and asked them for their consent to participate in the study.

The questionnaire was pre-tested with 156 participants. The minimum sample for pre-testing was calculated by taking 10 percent of the total sample. Same method, inclusion criteria and sampling technique was followed for pre-testing and the sample of pre-testing was not included in the final sample size.

For data collection, telephonic calls were made using official P&SHD landline connections over a period of two weeks from 12 July to 26 July 2021. This was done to increase respondents’ willingness to accept calls and therefore increase the response rate of the study, because a call from an official government organization is considered safe and secure, as opposed to a number from a private network. If a person has given the wrong number or the number of a relative, efforts were made to trace the individual using the available contact number. Numbers not responding on a specific day and time were re-attempted on other days at different times. If the telephone calls were not answered after three attempts on varying days and times, it was marked as non-response.

### 2.4. Training of Research Team

A one-day training was imparted to the research team. The interviewers were trained to communicate effectively with respondents to maximize responses. They were trained to answer potential questions of respondents (especially reluctant respondents) in a quick and confident manner to improve the response rate of the study. The research team was made aware of the contents of the survey tool, telephonic interview protocols, steps for implementing the survey tool, data submission guidelines, and roles and responsibilities of enumerators. They were also trained in following ethical considerations including respecting consent, anonymity, and confidentiality of the respondents. If the respondents were not willing to partake in the interview, they would not be forced to participate.

### 2.5. Ethical Considerations

The study protocols were reviewed and approved by the Institutional Ethics Review Board, University of the Punjab (D/No: 182/DFEMS/PU). The study strictly followed international guidelines for ethical review of epidemiological studies [30]. As described earlier, informed consent was obtained from all respondents over the telephone before formally starting the interview. The study was introduced to the respondents, and they were ensured of confidentiality of information and protection of privacy. They were guaranteed that their names and data, including health information, would only be used for research purposes.

Data collected by enumerators was submitted to field supervisors on a daily basis. All questionnaires received from the enumerators were checked by supervisors and data analysts before data entry. Data entry was done by data entry clerks, and to minimize the inconsistencies and human error, data was thoroughly cleaned by data analysts, data entry clerks, and field supervisors. Close coordination with the team lead and the deputy team lead of the project team for review and feedback was maintained throughout the project.

### 2.6. Statistical Analysis

Data entry was done in SPSS (version 26) (IBM, Armonk, NY, USA) by data entry clerks in parallel with data collection. After complete data entry, data was cleaned thoroughly by data entry clerks, data analysts, and field supervisors. After proper cleaning of data, it was analyzed using descriptive and inferential statistics in SPSS by data analysts. For the descriptive analysis, univariate analysis including frequencies, percentages, and graphs were used while for inferential statistics, Chi-square and binary logistic regression analysis with 95% confidence interval and 0.05 significance was used. The variables significant at binary logistic regression analysis were placed in multivariate logistic regression analysis. Odds ratio (OR), adjusted odds ratio (AOR) and 95 percent confidence intervals are presented. *p*-value less than 0.05 was considered statistically significant. In multivariable logistic regression, all independent variables that were significant at 0.05 level were entered in the model. The multi-collinearity between the variables was also assessed and highly correlated variables were eliminated from the logistical model. For example, multicollinearity was assessed between employment status and sector of employment. Since it was significant, sector of employment was eliminated from the model.

## 3. Results

### 3.1. Socio-Demographic Profile

The survey had 1325 respondents. The study found that a majority of the respondents were male (73%), in the age group of 40–49 (49%), residing in urban areas (78%), and Muslim in faith (95%) with a monthly income between 20,001–50,000 Pakistani rupees (PKR) (45%). A majority (94.05%) of the respondents were married and about half reported to have 3–4 children. Overall, a majority of the respondents (83.1%) had some level of education, with 26% having a higher level of education (Table 1). A majority of the respondents (65.1%) were employed. Overall, 69% respondents worked in the private sector and 31% worked in the public sector. About one-third (33%) of employed respondents were self-employed, while 21% were unskilled workers (such as delivery boy, driver, factory worker, waiter, house maid, security guard, tailor, sweeper, chef, shopkeeper, salesman, gardener, etc.) (see Table 1).

Table 1 presents the socio-demographic characteristics associated with the vaccination status of the respondents. A total of 14.3% of the vaccinated respondents and 28.9% of non-vaccinated group were from rural areas. The male sex (*p* = 0.000), 50 years and above age group (*p* = 0.04), income above PKR 20,000 (*p* = 0.05), marital status (*p* < 0.000), and number of children (*p* < 0.000) were variables that were significantly associated with the vaccination status of respondents (Chi-square, *p* < 0.05). Only 8.7% of vaccinated respondents had no education, compared to almost one-third of non-vaccinated respondents (29.2%). This shows greater vaccine acceptance in literate people (*p* < 0.000). Around 69.6% of vaccinated respondents were employed and nearly half of them (44.8%) were working in public sector. The employment status, sector of employment, family monthly income, and occupation were significantly (*p* < 0.000) associated with the vaccination status.

### 3.2. Access to Mass Media and Association with Vaccination Status

**Figure 1:**
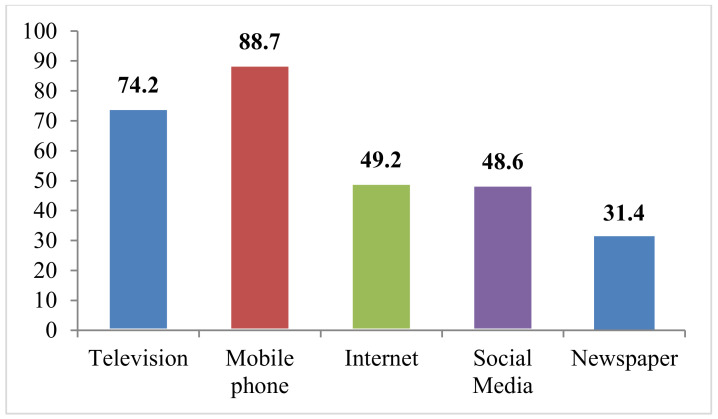
Percentage distribution of respondents’ access to media (*n* = 1317).

Vaccine hesitancy or acceptability is also based on knowledge of people regarding vaccine benefits. Therefore, questions were also included regarding people’s access to mass media to get COVID-19-related information. The findings revealed that mobile phone was the most common and widely used source of information as 88.7% of respondents had access to cellular devices. On the other hand, only 31.4% of respondents read the newspaper to get information, making it the least accessed source of mass media. Furthermore, a majority of the respondents (36.3%) had moderate access to mass media (2–3 sources of information), while 1.2% had high access (four sources of information) and 3.2% had no access to any source of information (Figure 1).

The study found that more vaccinated respondents had access to four sources of information compared to non-vaccinated respondents (48% vs. 34%). About 92.8% of vaccinated respondents had mobile phones compared to 82.6% of non-vaccinated respondents. Newspaper was the least used source of media for seeking information in both groups (vaccinated 38.7% and non-vaccinated 20.6%). Overall, access to media was significantly associated with the vaccination status of respondents (*p* = 0.000) (Table 2).

### 3.3. History of Any Medical/Health Condition and Allergies and Association with Vaccination Status

**Figure 2:**
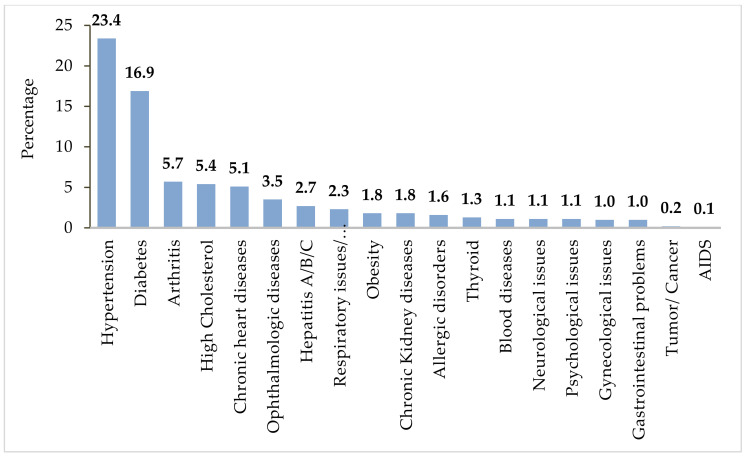
Percentage of history of different chronic conditions (*n* = 1325).

It can be seen in Figure 2 that 45.9% of respondents reported history with and/or presence of one or more chronic illness. About 23.4% of respondents had a history of hypertension followed by diabetes (16.9%), and 0.2% and 0.1% of respondents reported to have any kind of tumor/cancer and AIDS, respectively.

Contrary to other studies, the present research found that people with no history of chronic disease were in more favor of vaccination compared to those who did have such a history. The history of any medical health conditions was significantly associated with vaccination status (*p* = 0.05) (Table 3). People with any type of allergy (food, medicine, and vaccination) may be more susceptible to allergy with other drugs and/or vaccination than people with no allergy. About 3.8% respondents reported history of allergies with specific kind of medicines, 2.4% with any kind of food, 1% with any vaccination, and 1.4% with dust or cosmetics.

Respondents who reported allergic reactions with any food, medicine, and/or vaccination were then recoded as ‘history of any allergy‘, while respondents who did not report any history of allergies was recoded as ‘no history of allergy’. Overall, 8% of respondents had a history of allergies to any allergens (medicine/food/vaccination/dust/cosmetics). Similarly, about a half of the respondents who had a history of allergy reported a history of adverse event from these allergens. Around 92.1% of vaccinated respondents and 91.9% of non-vaccinated respondents did not have any allergies. It shows that almost a similar percentage of people reported history of any allergy in both the vaccinated and non-vaccinated groups (7.9% vs. 8.1%) (Table 3).

### 3.4. Refusal to Any Vaccination and History of Influenza Vaccine in the Past

Findings show that 4.8% of respondents reported they had refused any prior vaccination for themselves and/or their children. Furthermore, 20.3% the respondents had received influenza vaccination in the past. Table 4 depicts that 24.2% of vaccinated and 14.5% non-vaccinated respondents had received the influenza vaccine. The status of influenza vaccine received was statistically significant between the two study groups, however, refusal of any vaccination in the past was not proven to be related to the vaccination status of respondents (Table 4).

### 3.5. History of COVID-19 Virus Contraction and Association with Vaccination Status

History of COVID-19 virus contraction is important when it comes to analyzing the vaccination status. Overall, 7.5% of respondents had contracted COVID-19 in the past (Table 5). However, only 42.4% of these respondents had confirmed this by laboratory PCR test. No respondents reported the confirmation of COVID-19 infection by antibodies test. About half of the respondents (47.5%) suffered mild symptoms of COVID-19 infection while 8.1% had severe symptoms. Moreover, no respondents reported very serious symptoms of COVID-19 infection that led to hospitalization (Table 5). It can be seen that 8.2% of vaccinated respondents and 6.4% of non-vaccinated respondents had contracted COVID-19 in the past. No significant association was observed between vaccinated and non-vaccinated groups with respect to prior contraction of COVID-19.

### 3.6. Lifestyle and Self-Reported Health Status

Evidence has shown that smokers are more vulnerable to the damage caused by COVID-19 than non-smokers. About 25% of vaccinated and 28.5% of non-vaccinated respondents reported of cigarette smoking. No significant association was observed between the two groups. On the basis of previous findings, it was assumed that a person with healthy behaviors would be more in favor of vaccination as compared to someone with unhealthy behaviors. Table 6 shows that 63.2% of respondents reported that they performed any physical activity, which mostly included walking. About 43.2% of vaccinated respondents versus almost one-fourth, or 27.4%, of non-vaccinated respondents performed any type of physical activity. Physical activity was thus significantly associated with the vaccination status of respondents (*p* < 0.000) (Table 6). Results show that a majority of respondents (66.6%) ranked their health as ‘good’, while only 6.1% ranked their health as ‘poor’. A total of 68.9% of vaccinated and 63.2% of non-vaccinated respondents perceived their current health as ‘good’. Self-reported health status was therefore statistically significantly associated with vaccination status (*p* < 0.000) (Table 6).

### 3.7. Factors Encouraging and Discouraging People to Get Vaccination

Effectiveness of inoculation campaigns for curbing the spread of the pandemic is not only dependent on the safety and efficacy of the vaccine, but on its availability and attitudes of people [18]. Therefore, data on encouraging and discouraging factors was also collected. This section covers the factors that influenced respondents to get vaccinated or not get vaccinated.

### 3.8. Decision/Reasons to Get Vaccination

About a half (42.7%) of respondents reported that they were not influenced by anyone, and that it was their own decision to get vaccinated. About 32.4% reported to be influenced by their employer/colleagues/organization to get vaccination (Table 7).

**Figure 3:**
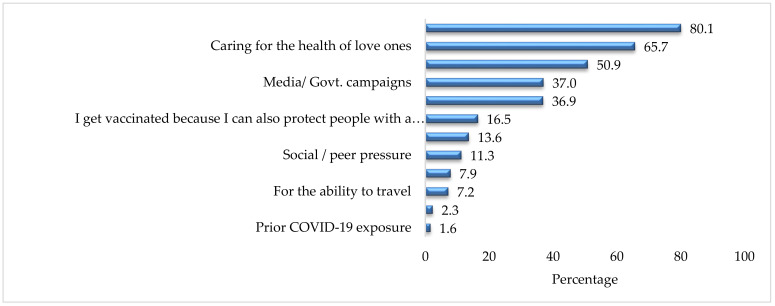
Reasons to get vaccination (%) (*n* = 794).

Factors that encouraged respondents to get vaccinated are provided in Figure 3. It can be seen that the majority (80.1%) of respondents who received the vaccine were concerned about their own health and the heath of their loved ones (65.7%). Almost a half of respondents (50.9%) were influenced by government campaigns and media campaigns (37.0%). Only 2.3% and 1.6% of vaccinated individuals reported they got vaccinated because of the presence of any chronic condition and prior COVID-19 exposure, respectively.

### 3.9. Decision/Reasons Not to Get Vaccination

Table 8 shows the factors that discouraged respondents to get vaccinated. A majority (75.3%) of non-vaccinated respondents reported that it was their own decision to not get the vaccine. Around 4.5% were advised by their doctor not to get vaccinated due to the presence of any chronic health conditions.

**Figure 4:**
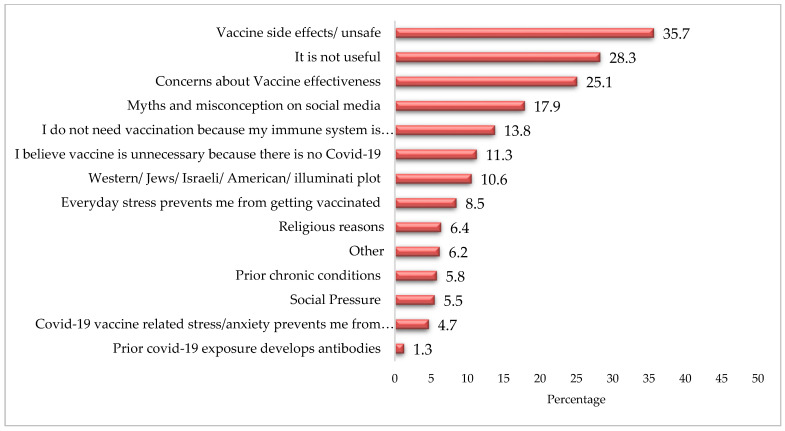
Factors discouraging getting vaccination (%).

Figure 4 shows that more than one-third (35.7%) of non-vaccinated respondents felt that the vaccine was unsafe and 28.3% believed that it was not useful. Moreover, one-fourth (25.1%) of respondents were concerned about vaccine effectiveness.

### 3.10. Logistic Regression Analysis for the Predictor Associated with Vaccinated Group

The results of the simple binary logistic regression show that the age group of 60–69 (OR = 1.52; 95% CI: 1.08–2.13; *p*-value: 0.02), middle (OR = 5.06; 95% CI: 3.59–7.15; *p*-value: 0.00), secondary (OR = 4.46; 95% CI: 2.91–6.85; *p*-value: 0.00) and higher (OR = 5.06; 95% CI: 3.51–7.29; *p*-value: 0.00) level of education, marital status (OR = 3.00; 95% CI: 1.85–4.85; *p*-value: 0.00), employment (OR = 1.63; 95% CI: 1.29–2.05; *p*-value: 0.00), family monthly income between PKR 20,001 and 50,000 (OR = 1.40; 95% CI: 1.07–1.83; *p*-value: 0.02), urban residence (OR = 2.50; 95% CI: 1.90–3.29; *p*-value: 0.00), high access to media (OR = 2.09; 95% CI: 1.11–3.92; *p*-value: 0.02), received influenza vaccine in the past (OR = 1.88; 95% CI: 1.41–2.50; *p*-value: 0.00), physical activity (OR = 2.02; 95% CI: 1.59–2.56; *p*-value: 0.00), and fair (OR = 2.56; 95% CI: 1.56–4.21; *p*-value: 0.00) and good (OR = 2.78; 95% CI: 1.73–4.45; *p*-value: 0.00) self-reported health status were significantly associated with the vaccination status of respondents (Table 9).

After adjusting age group, education level, monthly family income, respondents belonging to urban areas (AOR = 1.89; 95% CI: 1.36–2.63; *p*-value: 0.00), and employment status (AOR = 1.57; 95% CI: 1.17–2.11; *p*-value: 0.00) showed higher odds of getting vaccination. Similarly, respondents who received influenza vaccination were 1.8 times more likely to get vaccination (AOR = 1.81; 95% CI: 1.29–2.54; *p*-value: 0.00) than those who had not received this vaccination. Chances of getting vaccination were higher for those who were involved in any type of physical activity (AOR = 1.61; 95% CI: 1.23–2.13; *p*-value: 0.00) and with fair (AOR = 2.40; 95% CI: 1.28–4.48; *p*-value: 0.01) or good (AOR = 2.21; 95% CI: 1.20–4.06; *p*-value: 0.01) self-reported health status (Table 9).

## 4. Discussion

The best strategy to deal with any pandemic and to reduce the impact of infection is to ensure the availability of an effective and accessible vaccination. Likewise, to control the effects of the COVID-19 pandemic on people’s lives, the entire world is in the process of mass inoculations, with efforts to vaccinate a large proportion of the population in the shortest possible time, making it the fastest vaccine delivery in history [31]. However, as of now, only one-fourth of the total world’s population has been vaccinated [32]. The share of the vaccinated population varies from country to country with UAE having the highest vaccination rate of almost 90% being fully vaccinated while only 23.5% of the population of Pakistan is fully vaccinated, as of 3 December 2021 [32]. The varying vaccination rates in different countries can be attributed to hesitancy [7] and concerns of people regarding the effectiveness and safety of the vaccines [13].

This is the first province-wide study conducted in the province of Punjab examining the reasons for vaccine acceptance and hesitancy. Our study found that a majority of respondents were male, in the 40–49 age group, lived in urban areas, followed Islam as a religion, and belonged to the middle-income class. A relative low participation of women in the vaccination campaign can be seen within the context of socio-cultural aspects of Pakistani society. Women are socially and economically disadvantaged, which subjects them to various cultural restrictions including mobility constraint [33], vaccine registration of female respondents from the telephone number of any male member of family, hesitancy of females to talk to strangers or to give them any kind of information and relatively less possession of personal mobile devices by females compared to males [34], which makes women non-uniform passive recipients of benefits [35].

In addition to this, women in Pakistan are not majorly part of a formal workforce of the country and they do not have any obligation of getting vaccinated, as a majority of the females resides at home as housewives [33]. It is important to mention here that workplaces in Pakistan, especially in the public sectors, have made it compulsory for their employees to get COVID-19 vaccination [36].

The findings of the present study are consistent with previous research. For example, socio-demographic variables such as age group being 60–69 years [37,38,39,40,41,42,43,44], middle or higher level of education [38,40,41,42,45,46,47], being currently married [48,49,50], being employed [42,51,52], having a monthly family income of 20,001–50,000 PKR [40,43,45,47,48,51], residing in urban areas, having high access to mass media, having a history of influenza vaccination [38,41,49,53,54,55], performing physical activity, and perceiving fair and good status of health [47], were significantly associated with COVID-19 vaccination uptake. Findings revealed that the pandemic had largely affected the income, work, and daily lives of more than 40% of the respondents. Despite this, people feel reluctant to get vaccinated. Low vaccination coverage, despite the Government’s best efforts, may point towards the hesitancy and reluctance of people in accepting the vaccines [7]. In order to expand vaccination coverage and to achieve maximum vaccination rates, understanding people’s perception and behavior towards vaccines is necessary.

There are different reasons that contribute towards increasing people’s reluctance to accepting vaccines in general, including concerns about vaccine effectiveness, and its safety and side effects [14]. However, in a country such as Pakistan, vaccine acceptance rates are low as the health decisions of a majority of the population is also influenced by various religious, cultural, social, and ethnic values [9,10,11]. Similar to our study, various studies from Pakistan show that people in Pakistan perceive vaccination campaigns to be a Western/American/Illuminati agenda [9], as an attempt of genocide against Muslims [11] and/or a means to reduce the fertility rates of Muslims [10]. Greater vaccine hesitancy rates in Pakistan also points towards the lack of health literacy and lack of trust of government policies [12]. In addition to this, there are various myths and misinformation attached with the COVID-19 vaccine. For example, a large proportion of the non-vaccinated respondents believed that the vaccines were unsafe (35.7%); moreover, 28.3% considered it useless, while one-fourth (25.1%) of the respondents reported to be concerned about vaccine effectiveness.

Given the current situation of the healthcare system in Pakistan, and previous experiences of different vaccination campaigns, understanding vaccine acceptance in Pakistan is very necessary to control the pandemic. The attitudes and perceptions of people regarding vaccines are key factors that determine vaccine acceptance [18]. Pakistan, being a country of rich cultural and religious profiles, has cultural variations that are prevalent in the shape of ethnicity as well as based on region of residence (rural/urban/semi urban) [56]. This study found that people believed in the emergence of Islamophobia in the West, due to which they perceived the COVID-19 infection as well as the vaccination campaign as a genocidal attempt towards Muslims to control their population by causing infertility and considering it a “Western plot” in general, as is perceived about other vaccination campaigns by some communities in Pakistan [9,10,11]. Additionally, the prevalence of other myths and misconceptions included considering COVID-19 to be a man-made virus [18,57], fear of insertion of a secret micro-chip under the guise of vaccination [18], increased chances of death, and a lack of trust in the government [40], health workers [50], and the vaccine [24,39,42,47,51,58,59,60]. The present study reported these factors to be responsible for vaccine hesitancy in Pakistan. Other factors highlighted by the present study that contributes to COVID-19 vaccine hesitancy among people include the perceived side effects of the COVID-19 vaccine [15,24,38,52,53,60,61,62], concerns of people regarding different types of vaccines developed in a short period of time [58,60], and their alleged side effects including increased chances of death. People also reported some structural barriers, such as time constraint and facility accessibility issues [19,48]. This highlights the need for developing strategies to not only eliminate attitudinal but structural barriers as well.

In accordance with the findings of previous studies [24,39], the present study revealed that females are more hesitant than males in getting COVID-19 vaccination while people working in the private sector, had any chronic illness, were inoculated with influenza vaccination in the past or had contact with COVID-19 cases were more likely to accept the vaccine. Fisher et al. [38] also revealed the higher likelihood of influenza vaccine recipients to accept the COVID-19 vaccine in the United States while Reno and colleagues [52] reported the positive association of the prevalence of any chronic illness with the likelihood of getting the COVID-19 vaccination. The present study further reported that people with higher levels of education and income were more likely to accept the COVID-19 vaccine, which also echoes the findings in the literature on COVID-19 vaccine acceptance [38,52].

The results of the present study as well as of many previous studies that explored the reasons for vaccine hesitancy highlighted that the majority of unvaccinated people did not want to get vaccinated due to a low perceived susceptibility or risk to contracting the virus, believe in the conspiracies against the vaccine [43,57], religious reasons [10,11] and concerns about the efficacy, effectiveness, and safety of the vaccine [14]. There are rigorous COVID-19 vaccination campaigns being implemented globally including Pakistan. These campaigns have met with some success by targeting a large number of the population. However, more comprehensive and concerted efforts such as Reach Every Door and Reach Every Community (RED/REC) are needed to engage the community in the vaccination campaign to understand as well as tackle attitudinal barriers, especially in the context of Pakistan that is a milieu of various socio-cultural, ethnic, and religious forces and lags far behind in achieving the goal of Universal Health Coverage [63].

## 5. Limitations and Strengths of the Study

As expected in telephonic surveys, the limitation of the present study is selection bias. This survey may have excluded the digitally illiterate people who do not possess a personal cell phone and got their vaccinations registered using someone else’s number. However, in such cases, efforts were made to reach to the registered person by requesting the person to provide the number of any family member of the registered person. Another limitation could be information bias, particularly providing socially desirable answers. The questions were asked by trained enumerators and the tools were pre-tested on a sample of 156 respondents, so efforts were made to overcome these biases. Additionally, the data on vaccination status was obtained from the Primary and Secondary Health Department in addition to asking questions from the respondents.

In addition to this, this survey is more likely to have acceptance rate from those who were motivated to complete this and were more likely to have stronger opinions related to the COVID-19 vaccination, attracting selection bias. Additionally, on the other hand, the strengths of the present study include exploring people’s hesitancy or acceptance of the COVID-19 vaccination as well as its association with demographic factors to predict COVID-19 vaccine uptake. The results of this study provide policy makers and key shareholders an insight into how to effectively target public health campaigns.

## 6. Conclusions

The study concludes that there are various socio-economic characteristics such as sex, income, education, employment, rural residence, as well as access to sources of information, history of influenza vaccination, and disease-related factors which were associated with COVID-19 vaccine uptake. However, vaccine non-recipients were influenced by many myths and misinformation which hindered them in getting vaccinated. These myths, misinformation, and conspiracy theories need to be addressed individually and systematically through effective and well-targeted information, education, and communication strategies. Because of attitudinal barriers, people have a lack of trust in the government, healthcare workers, and on vaccine efficacy and effectiveness. Multi-faceted, comprehensive approaches are needed which involve the entire population and are tailored for different groups, especially for marginalized sections of the society. Behavioral change interventions involving all relevant stakeholders at individual, community, and institutional levels are needed to deal with the barriers perceived by people. In order to expand vaccination coverage and improve vaccine acceptance in Pakistan, academics, public health experts, policy makers, and healthcare workers need to work together to enhance community participation and community mobilization for the COVID-19 vaccination program.

## Figures and Tables

**Table 1:** Socio-demographic status of respondents by their vaccination status (*n* = 1325).

Characteristics	Vaccinated		Not Vaccinated		Total		*p*-Value ^e^
	Frequency	%	Frequency	%	Frequency	%	
Sex (*n* = 1325)							
Male	626	78.7	346	65.3	972	73.4	0.00
Female	169	21.3	184	34.7	353	26.6	
Age group (*n* = 1325)							
40–49	367	46.2	279	52.6	646	48.8	0.04
50–59	265	33.3	156	29.4	421	31.8	
60–69	128	16.1	64	12.1	192	14.5	
70–79	29	3.6	23	4.3	52	3.9	
80+	6	0.8	8	1.5	14	1.1	
Mean age ± SD	51.7 ± 9.19		50.4 ± 9.52		51.2 ± 9.34		
Area of Residence (*n* = 1318) ^a^							
Rural	113	14.3	153	28.9	266	20.2	0.00
Urban	664	84.3	360	67.9	1024	77.7	
Semi Urban	11	1.4	17	3.2	28	2.1	
Region (*n* = 1319) ^a^							
Central Punjab	596	75.0	464	88.5	1060	80.4	0.00
Northern Punjab	147	18.5	34	6.5	181	13.7	
Southern Punjab	52	6.5	26	5.0	78	5.9	
Religion (*n* = 1322) ^a^							
Islam	754	95.2	516	97.4	1270	96.1	0.04
Christianity	37	4.7	14	2.6	51	3.9	
Others	1	0.1	0	0	1	0.1	
Marital Status (*n*= 1312) ^a^							
Currently married	757	96.6	477	90.3	1234	94.1	0.00
Not currently married ^b^	27	3.4	51	9.7	78	5.9	
No. of children (*n* = 1257) ^a^							
0	23	3.1	17	3.4	40	3.2	0.00
1–2	162	21.5	127	25.1	289	23.0	
3–4	400	53.2	203	40.2	603	48.0	
5 and above	167	22.2	158	31.3	325	25.9	
Monthly family income in Pakistani Rupees (PKR) ^c^ (*n* = 1173) ^a^							
≤20,000	184	27.9	172	33.5	355	30.3	0.05
20,001–50,000	314	47.6	210	40.9	525	44.7	
50,001–100,000	116	17.6	85	16.5	201	17.1	
Above 100,000	45	6.8	47	9.1	92	7.8	
Education level (*n* = 1317) ^a^							
No formal education	69	8.7	154	29.2	223	16.9	0.000
Primary (1–5)	42	5.3	67	12.7	109	8.3	
Middle (6–10)	329	41.6	145	27.5	474	36.0	
Secondary (11–12)	112	14.2	56	10.6	168	12.8	
Higher (13 and above)	238	30.1	105	19.9	343	26.0	
Employment status (*n* = 1311)							
Employed	544	69.6	309	58.4	853	65.1	0.00
Not employed ^d^	238	30.4	220	41.6	458	34.9	
Sector of employment (*n* = 848)							
Public	242	44.8	21	6.8	263	31.0	
Private	298	55.2	287	93.2	585	69.0	
Nature of occupation (*n* = 825)							
Self–employment/Business	89	17.0	188	62.0	277	33.6	
Doctor/Health Worker	24	4.6	3	1.0	27	3.3	
Teacher/Professor/Educator	44	8.4	13	4.3	57	6.9	
Police Department	23	4.5	4	1.3	27	3.3	
Professional/Managers	58	11.1	7	2.3	65	7.9	
Field worker	60	11.5	31	10.2	91	11.0	
Clerical/Official staff	66	12.6	3	1.0	69	8.4	
Skilled	34	6.5	5	1.7	39	4.7	
Unskilled	124	23.8	49	16.2	173	21.0	

^a^ The data is not 100% accurate because of missing values. ^b^ Includes widow/widower, divorced, separated and single. ^c^ 1 USD = 165 PKR. ^d^ Includes people who were not working currently, housewives and retired). ^e^ *p*-value was calculated on the basis of X^2^ statistics.

**Table 2:** Access to sources of information.

Characteristics	Vaccinated		Not Vaccinated		Total		*p*-Value
	Frequency	%	Frequency	%	Frequency	%	
Level of exposure (*n* = 1317)							
No access	21	2.7	21	4.0	42	3.2	0.00
Low access *	106	13.5	129	24.4	235	17.8	
Moderate access **	281	35.7	197	37.2	478	36.3	
High access ***	380	48.2	182	34.4	562	42.7	

* access to one media source, ** access to two or three media sources, *** access to four or more sources, *p*-value was calculated on the basis of X^2^ statistics.

**Table 3:** History of any medical/health condition and any allergy among respondents (*n* = 1325).

Characteristic	Vaccinated		Not Vaccinated		Total		*p*-Value
	Frequency	%	Frequency	%	Frequency	%	
History of any medical and health condition (*n* = 1325)							
Respondents with no disease	448	56.4	269	50.8	717	54.1	0.05
Respondents with any chronic disease	347	43.6	261	49.2	608	45.9	
History of any allergy (*n* = 1325)							
Yes	63	7.9	43	8.1	106	8.0	0.90
No	732	92.1	487	91.9	1219	92.0	

The *p*-value was calculated on the basis of X^2^ statistics.

**Table 4:** Vaccination history of respondents.

Characteristics	Vaccinated		Not Vaccinated		Total		*p*-Value
	Frequency	%	Frequency	%	Frequency	%	
Refused any vaccination in the past for their children and themselves (*n* = 1322)							
Yes	35	4.4	28	5.3	63	4.8	0.46
No	758	95.6	501	94.7	1259	95.2	
Received vaccination against Influenza (*n* = 1323)							
Yes	192	24.2	77	14.5	269	20.3	0.00
No	601	75.8	453	85.5	1054	70.7	

The *p*-value was calculated on the basis of X^2^ statistics.

**Table 5:** History of COVID-19 virus contraction.

Characteristics	Vaccinated		Not Vaccinated		Total		*p*-Value
	Frequency	%	Frequency	%	Frequency	%	
COVID-19 positive in the past (*n* = 1325)							
Yes	65	8.2	34	6.4	99	7.5	0.23
No	730	91.8	496	93.6	1226	92.5	
Confirmation of COVID-19 infection (*n* = 99)							
Not confirmed, suspected	22	33.8	19	55.9	41	41.4	
Yes, confirmed by doctor upon symptoms	10	15.4	6	17.6	16	16.2	
Yes, confirmed by test	33	50.8	9	26.5	42	42.4	
Yes, confirmed by antibodies test	0	0.0	0	0.0	0	0.0	
Severity of COVID-19 infection (*n* = 99)							
Asymptomatic	7	10.8	2	5.9	9	9.1	
Mild	31	47.7	16	47.1	47	47.5	
Moderate	21	32.3	14	41.2	35	35.4	
Severe	6	9.2	2	5.9	8	8.1	
Very Heavy	0	0.0	0	0.0	0	0.0	

**Table 6:** Life style and self-reported health status.

Characteristics	Vaccinated		Not Vaccinated		Total		*p*-Value
	Frequency	%	Frequency	%	Frequency	%	
Do you do any physical activity (*n* = 1322)							
Yes	342	43.2	145	27.4	487	36.8	0.00
No	450	56.8	385	72.6	835	63.2	
Self-reported health status (*n* = 1324)							
Poor	30	3.8	51	9.6	81	6.1	0.00
Fair	217	27.3	144	27.2	361	27.3	
Good	547	68.9	335	63.2	882	66.6	

The *p*-value was calculated on the basis of X^2^ statistics.

**Table 7:** Decision to get vaccination (*n* = 794).

Respondent Influenced to Get Vaccination by	Vaccinated	
	Frequency	%
Family	200	25.2
Employer/colleagues/organization	257	32.4
Doctor	32	4.0
COVID-19-infected people among family/friends	26	3.3
No one	339	42.7
Any other	3	0.3

**Table 8:** Decision/reasons not to get vaccination (*n* = 530).

Who Influenced Not to Get Vaccination	Not Vaccinated	
	Frequency	%
Friends	32	6.0
Family	85	16.0
Employer/colleagues/organization	3	0.6
Doctor	24	4.5
COVID-19 infected people among family/friends	10	1.9
No one	399	75.3
Any other	22	4.2

**Table 9:** Bivariate and multivariable logistic regression for factors associated with vaccination status of respondents (n = 1325).

Variable	OR	95% CI	*p*-Value	AOR	95% CI	*p*-Value
Age group (*n* = 1325)						
40–49	1					
50–59	1.29	(1.00–1.66)	0.05	1.52	(1.12–2.05)	0.01
60–69	1.52	(1.08–2.13)	0.02	2.44	(1.61–3.71)	0.00
70–79	0.96	(0.54–1.69)	0.88	1.49	(0.69–3.23)	0.31
80+	0.57	(0.20–1.66)	0.30	1.96	(0.55–6.93)	0.30
Education level (*n* = 1325)						
No education	1					
Primary	1.40	(0.87–2.26)	0.17	1.43	(0.83–2.47)	0.19
Middle	5.06	(3.59–7.15)	0.00	4.64	(3.08–6.99)	0.00
Secondary	4.46	(2.91–6.85)	0.00	3.68	(2.17–6.23)	0.00
Higher	5.06	(3.51–7.29)	0.00	4.75	(2.85–7.90)	0.00
Monthly family income (*n* = 1173)						
≤20,000	1					
20,001–50,000	1.40	[1.07–1.83]	0.02	1.00	[0.73–1.37]	0.99
50,001–100,000	1.28	[0.90–1.81]	0.17	0.60	[0.39–0.92]	0.02
Above 100,000	0.90	[0.57–1.42]	0.64	0.35	[0.20–0.61]	0.00
Region (*n* = 1318)						
Rural	1					
Urban	2.50	(1.90–3.29)	0.00	1.89	(1.36–2.63)	0.00
Semi Urban	0.88	(0.40–1.94)	0.74	0.46	(0.17–1.25)	0.13
Working status (*n* = 1311)						
Unemployed	1					
Employed	1.63	(1.29–2.05)	0.00	1.57	(1.17–2.11)	0.00
Marital Status (*n* = 1312)						
Not in relationship	1					
Married	3.00	(1.85–4.85)	0.00	1.71	(0.95–3.09)	0.07
Access to Media (*n* = 1317)						
No access	1					
Low access	0.82	(0.43–1.59)	0.56	0.49	(0.23–1.06)	0.07
Moderate access	1.43	(0.76–2.68)	0.27	0.69	(0.33–1.44)	0.32
High access	2.09	(1.11–3.92)	0.02	0.75	(0.35–1.61)	0.47
Disease status (*n* = 132)						
No disease	1					
Any chronic disease	0.80	(0.64–1.00)	0.05	0.90	(0.68–1.19)	0.45
Received influenza vaccine in past (*n* = 1322)						
No	1					
Yes	1.88	(1.41–2.50)	0.00	1.81	(1.29–2.54)	0.00
Have any physical activity (*n* = 1322)						
No	1					
Yes	2.02	(1.59–2.56)	0.00	1.61	(1.23–2.13)	0.00
Self-reported health status (*n* = 1324)						
Poor	1					
Fair	2.56	(1.56–4.21)	0.00	2.40	(1.28–4.48)	0.01
Good	2.78	(1.73–4.45)	0.00	2.21	(1.20–4.06)	0.01

Abbreviation: 1 is the reference category; OR, odds ratio; AOR, adjusted odds ratio; CI, confidence interval.

## Data Availability

The data presented in this study are available upon request from the corresponding author.
